# Development of a miniaturized 96-Transwell air–liquid interface human small airway epithelial model

**DOI:** 10.1038/s41598-020-69948-2

**Published:** 2020-08-03

**Authors:** Teresa Bluhmki, Sarah Bitzer, Julia Anna Gindele, Eva Schruf, Tobias Kiechle, Megan Webster, Jürgen Schymeinsky, Robert Ries, Florian Gantner, Daniel Bischoff, James Garnett, Ralf Heilker

**Affiliations:** 1 Department of Drug Discovery Sciences, Boehringer Ingelheim Pharma GmbH & Co. KG, Biberach an der Riß, Germany; 2 Department of Immunology and Respiratory Diseases Research, Boehringer Ingelheim Pharma GmbH & Co. KG, Biberach an der Riß, Germany; 3 Department of Cardiometabolic Diseases Research, Boehringer Ingelheim Pharma GmbH & Co. KG, Biberach an der Riß, Germany; 4 Department of Translational Medicine and Clinical Pharmacology, C. H. Boehringer Sohn AG & Co. KG, 88397 Biberach an der Riß, Germany

**Keywords:** Biochemistry, Cell biology, Drug discovery, Biomarkers

## Abstract

In order to overcome the challenges associated with a limited number of airway epithelial cells that can be obtained from clinical sampling and their restrained capacity to divide ex vivo, miniaturization of respiratory drug discovery assays is of pivotal importance. Thus, a 96-well microplate system was developed where primary human small airway epithelial (hSAE) cells were cultured at an air–liquid interface (ALI). After four weeks of ALI culture, a pseudostratified epithelium containing basal, club, goblet and ciliated cells was produced. The 96-well ALI cultures displayed a cellular composition, ciliary beating frequency, and intercellular tight junctions similar to 24-well conditions. A novel custom-made device for 96-parallelized transepithelial electric resistance (TEER) measurements, together with dextran permeability measurements, confirmed that the 96-well culture developed a tight barrier function during ALI differentiation. 96-well hSAE cultures were responsive to transforming growth factor β1 (TGF-β1) and tumor necrosis factor α (TNF-α) in a concentration dependent manner. Thus, the miniaturized cellular model system enables the recapitulation of a physiologically responsive, differentiated small airway epithelium, and a robotic integration provides a medium throughput approach towards pharmaceutical drug discovery, for instance, in respect of fibrotic distal airway/lung diseases.

## Introduction

A compromised barrier function of the airway epithelium is implicated in a panoply of respiratory pathologies. In the lung, the airway epithelium acts as both a physical and immunological barrier that protects the subepithelial tissue. The processes underlying the pathological breakdown of the epithelial barrier cannot be studied in the human lung outside of the clinical setting. For instance, animal models of fibrotic lung diseases^1,2^ such as the bleomycin model^2^ are only of restricted value since interspecies differences in regard to pulmonary anatomy, immune systems, fibrotic mechanisms and inflammatory responses are extensive. Furthermore, the use of animal experiments in pharmaceutical research has become more and more controversial for ethical reasons. In consequence, the pharmaceutical industry has been striving over decades to establish reliable, miniaturized in vitro models of the human lung epithelial cell layer for drug discovery in the area of respiratory diseases.

Earlier studies analyzed lung epithelial cells cultured under submerged conditions. In contrast, the Transwell format positions the in vitro epithelium on a synthetic membrane at the boundary between air and culture medium^3,4^, known as air–liquid interface (ALI). However, the commonly used large scale formats (e.g. 24-Transwell plates) hamper the application of ALI conditions to higher throughput profiling of drug candidates. Apart from the miniaturization of ALI cultures, also the provision of physiologically relevant lung epithelial cells in sufficient numbers is a challenge. Thus, several immortalized cell lines fail to reproduce several features of these disease relevant lung epithelial subregions. For instance, A549 cells, derived from human lung carcinoma^5,6^, may be passaged and cultured with relative ease, but they lack the pseudostratified organization of the bronchoepithelial layer^7^. On the other hand, the access to primary human bronchoepithelial cells is extremely limited and cost intensive. In order to study cystic fibrosis, for instance, a 96-Transwell format was explored using lung epithelial cells derived from larger airways^8^. However, assays employing human small airway epithelial (hSAE) cells^9^^,^ which are considered to be pathophysiologically relevant for respiratory diseases affecting the distal airways/lung including Idiopathic Pulmonary Fibrosis (IPF)^10–12^, have so far never been miniaturized to a similar extent.

hSAE cells are isolated from the distal part of the human lung^9,13,14^. After a few weeks of ALI culture they establish a pseudostratified layer of basal, club, goblet and ciliated cells that resembles the small airway epithelium as found in vivo ^3^. Basal cells provide a progenitor population, which can self-renew and is able to differentiate into ciliated, goblet and club cells. Thus, basal cells are essential to quickly repopulate the epithelium after damage^15^. Club cells are cubically shaped, secretory cells with uteroglobin (SCGB1A1, Secretoglobin Family 1A Member 1) being the primary secretory product^16^. They have several protective functions, including airway repair, secretion of anti-inflammatory and immunomodulatory proteins, and xenobiotic metabolism. Goblet cells are columnar cells that secrete mucins, mainly Mucin-5B (MUC5B) and Mucin-5AC (MUC5AC), towards the apical side of the epithelium^17^. In vivo, the resulting mucus traps airborne particles and is transported out of the lung by the ciliated cells^18^. Hence, all four cell types contribute to the proper functioning of the small airway epithelium, and it is important to include all of them into an in vitro disease model of IPF.

Transforming growth factor β1 (TGF-β1) is a key disease mediator in IPF^11,19^. Under healthy conditions, TGF-β1 signaling is important for morphogenesis during embryonic development and for tissue homeostasis. In the IPF lung, TGF-β1 promotes the release of other pro-inflammatory molecules and induces the secretion of extracellular matrix (ECM) components such as collagen or fibronectin^20^. Apart from TGF-β1, also tumor necrosis factor α (TNF-α) plays an important role in IPF pathogenesis^21^. In the small airway and alveolar microenvironment of the IPF lung, the pro-inflammatory cytokine TNF-α is expressed by macrophages and the epithelium itself. Like TGF-β1, TNF-α induces the release of other pro-inflammatory cytokines and chemokines such as monocyte chemoattractant protein 1 (MCP-1)^22^. MCP-1 has been shown to act as an important effector in IPF ^23^^,^ recruiting immune competent cells like macrophages to the inflammatory side and leading to a further activation of the recruited cells. Cytokines like TNF-α also induce an overproduction of matrix metalloproteinases (MMPs), such as MMP9^24^. The dysregulated MMPs play a central role in IPF, for instance, by contributing to the production of the characteristic fibrotic scars in IPF patients^25^.

In this work, a robotic ALI platform was established which enabled unattended, fully automated media exchange in 96-Transwell plates during a five-week maturation of primary hSAE cells first in submerged then in ALI culture. By automation and miniaturization, hSAE cells can be employed at higher throughput in an affordable, cost-effective manner. This technical innovation opens new routes towards target-focused and phenotypic drug discovery^26^.

## Results

### Semi-automated hSAE cell seeding and automated long-term maintenance of ALI cultures in 96-Transwell plates

**Figure 1:**
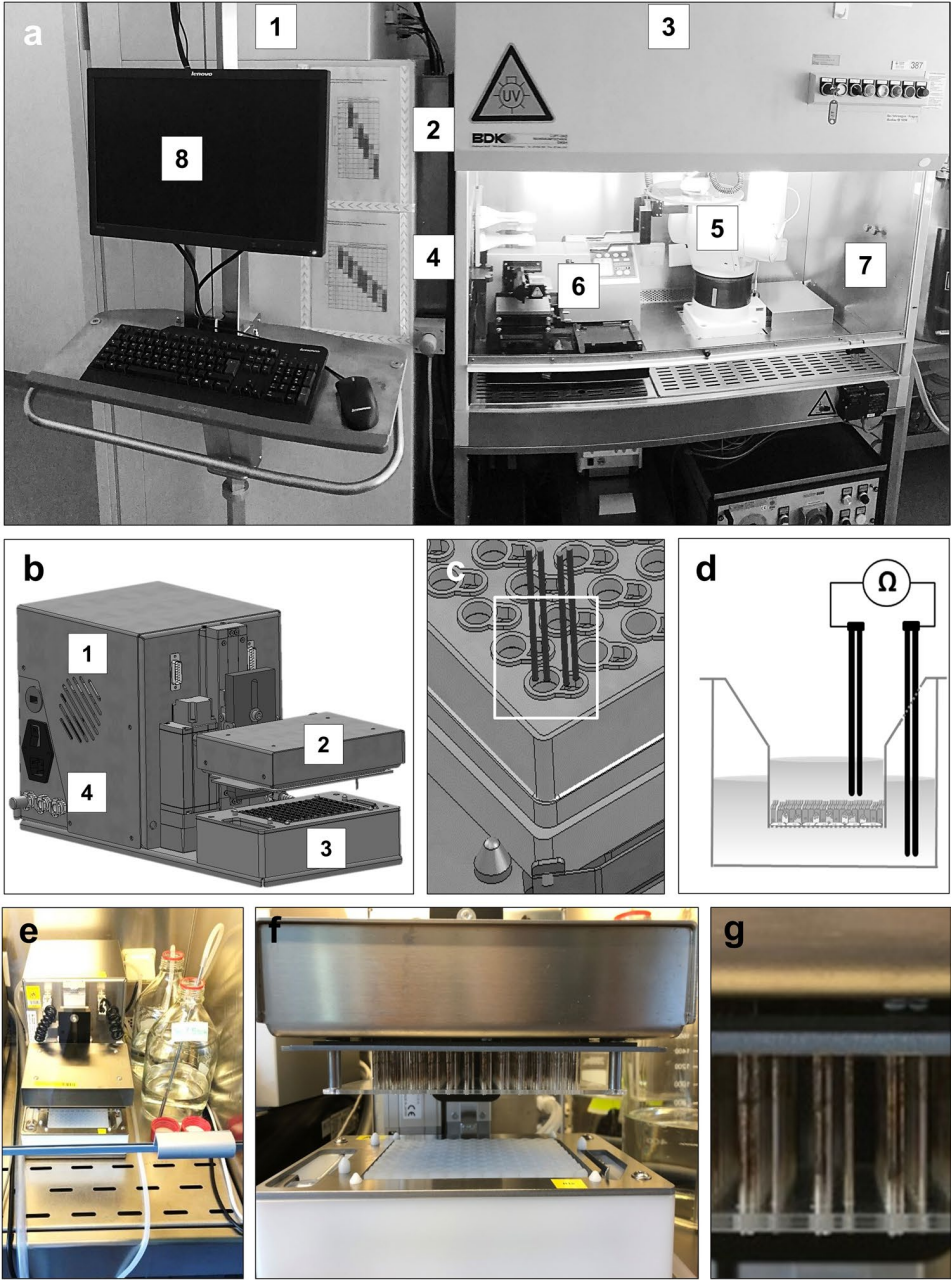
Semi-automated hSAE cell seeding and automated long-term maintenance of ALI cultures in 96-Transwell plates, a process overview. (**a**) hSAE cells from a frozen stock were thawed and resuspended in expansion medium, kept in suspension by gentle shaking of the falcon, and seeded into 96-Transwell plates using a Multidrop Combi device. Enabling of long-term maintenance of hSAE cells under ALI conditions in 96-Transwell plates via an integrated robotic system. Cell culture room was equipped with an HEPA-filtered air feed and ambient air overpressure. For plate maintenance, an automated microplate incubator (1) was combined with a laminar flow hood (3) via an airtight lock (2). A plate shuttle system (4) transported single 96-Transwell plates out of the incubator through the airtight lock into the laminar flow hood. Plates were collected from the airtight lock through a robotic arm (5). Inside the laminar flow hood a combined washer dispenser device (6) exchanged the medium from the Transwell plates, which was connected to a reservoir bag (7). Regular medium exchange on each plate was controlled and scheduled by a specialized software (8). (**b**) Representative overview of the customized constructed transepithelial electrical resistance (TEER) device. PC-controlled high-throughput TEER measurement for epithelial monolayer, including a data acquisition tool (1). The main components include: the robotic sampler, containing a panel of 96 individual electrodes, which moves the electrodes in each well of the Transwell plate (2), a base plate for the 96-well tray, including an electrode washing station (3), which is connected to different valves for waste, ethanol and sterile water. (**c**) Magnification of one single electrode pair, lowered in to the upper and basal compartment of the Transwell plate (Details of employed software see Supplementary Information, online). (**d**) Cross section of one single Transwell during a classical TEER measurement. (**e**) Overview of the actual device placed under the laminar flow hood. (**f**) Magnification of the robotic sampler, containing a panel of 96 individual (dual) electrodes (**g**) Magnification of the 96 individual electrode pairs per Transwell insert.

In order to enable higher throughput applications, several process steps in the preparation and maintenance of 96-Transwell plates with hSAE cells were automated. For this purpose, an integrated robotic system was developed. In order to meet the challenges of the required 5-week maturation and differentiation period of hSAE cells, the robotic system was set up in a cell culture room with HEPA-filtered air feed and ambient air overpressure. In this system (Fig. 1a), an automated cellular incubator for up to five hundred 96-Transwell plates was linked to a laminar flow hood via an airtight lock. Individual Transwell plates were moved within the robotic system using the plate shuttle system of the incubator in combination with a robotic arm taking over the microplate at the airtight lock between the incubator and the laminar flow. The robotic arm was programmed to place the Transwell plate onto a Biotek EL406 Dispenser washer device and to remove its lid. For the first phase of submerged Transwell culture, the Biotek washer exchanged the medium both in the upper and in the lower compartment of the Transwell plates. For the second phase of hSAE cell maturation under ALI conditions, the Biotek washer removed the medium from the upper compartments of the Transwell plates by aspiration, and continued to carry out a regular medium exchange in the lower compartment over the subsequent 4 weeks. A scheduling software applied the correct medium exchange protocol to the respective Transwell plates in the incubator at the appropriate time-points. For a detailed description of the upscaling and higher throughput adaptation of hSAE cell pre-culture, see Supplementary Figure S1 online.

A TEER device with an array of ninety-six dual electrode pairs was developed to analyze the integrity of the epithelial layers in every well of a 96-Transwell plate in parallel (Fig. 1b,e). For the TEER measurement, the upper compartments of the Transwell plate were transiently filled with ALI medium. Then, the electrode array was immersed into the upper and lower compartments of the Transwell plate (Fig. 1c–d, f–g). This technique allowed for quality control measurements across all 96 wells of the microplate in approximately one minute as described below. Apart from time saving, the rigid arrangement of the electrode array with a defined geometric positioning of the electrodes with respect to the epithelial layers granted a high degree of reproducibility for the TEER measurements. In the automated robotic system, the TEER values serve as a quality control of proper epithelial barrier formation during the ALI-conditioned differentiation process.

### Cellular composition of the hSAE cell-based epithelium in 96-Transwell format

**Figure 2:**
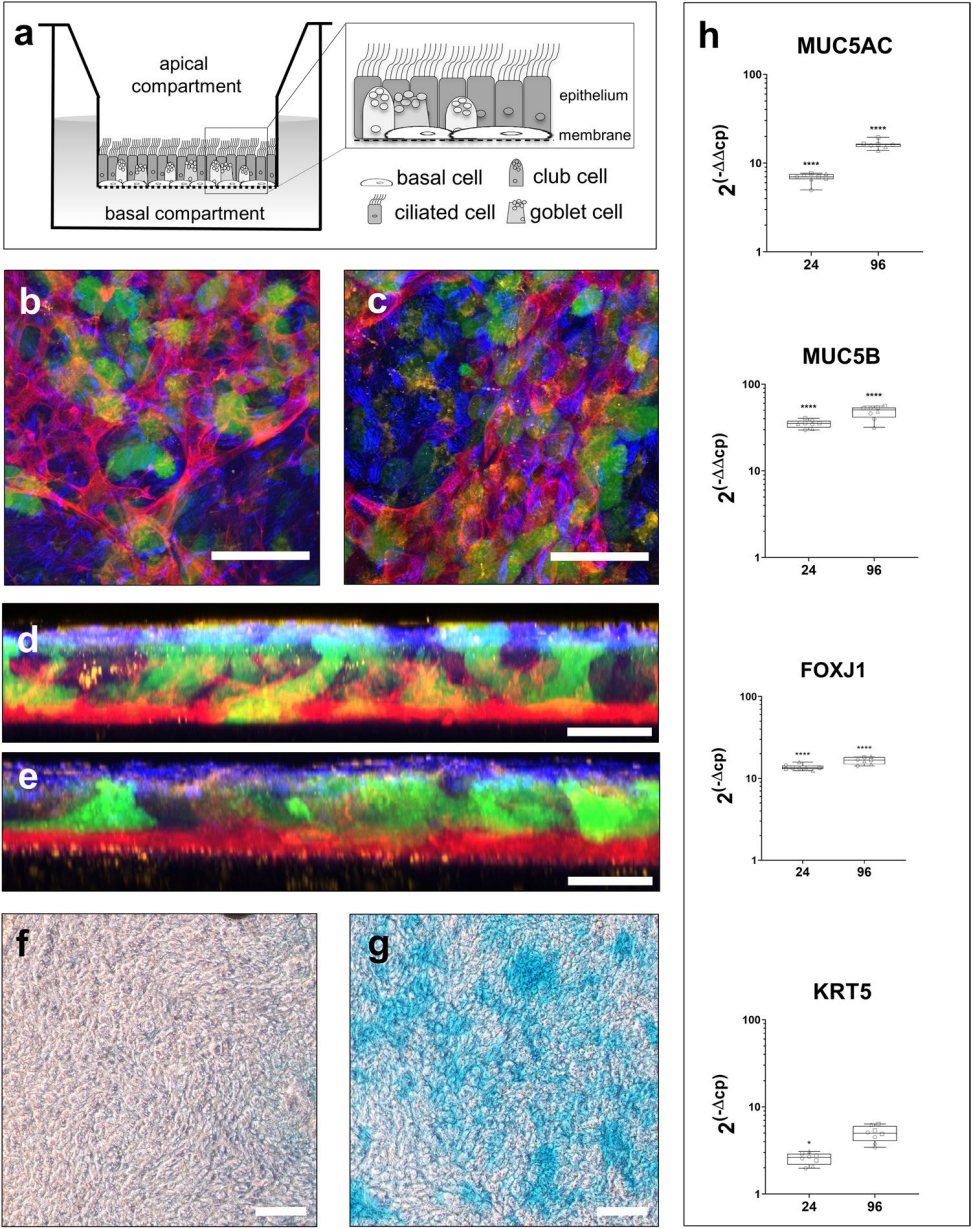
Characterization and comparison of small airway epithelial cell ALI cultures between 24- and 96-Transwell formats. (**a**) Schematic of small airway epithelium and their respective cell types, cultured in a single Transwell of a Transwell-plate (**b**, **c**) Confocal images (XY 3D) of the four different cell types present in small airway epithelial cell culture, cultured at the air–liquid interface after four weeks under either 24-Transwell (**b**) or 96-Transwell (**c**) conditions. KRT5 for basal cells (red), AcTubulin for ciliated cells (blue), MUC5AC for goblet cells (green) and SCGB1A1 for club cells (yellow). Scale bar = 50 µm (**d**, **e**) Images shown are representative of a high intensity projection (XZ 3D) of either 24-well culture (**d**) or the HTS adapted 96-well culture (**e**). Scale bar = 25 µm. (**f**) Microscopic images of the glycoprotein staining in the small airway epithelial cells after 1 week and (**g**) after 4 weeks of air–liquid interface culture in 96-Transwell plates. Scale bar = 200 µm. (**h**) Gene expression analysis of different small airway epithelial markers *MUC5AC* (*24:* N = 3, n = 8; *96*: N = 3, n = 7), *MUC5B* (*24:* N = 3, n = 8; *96*: N = 3, n = 8); *FOXJ1* (*24:* N = 3, n = 8; *96*: N = 3, n = 7); *KRT5* (*24:* N = 3, n = 8; *96*: N = 3, n = 8).

In larger Transwell formats, it has previously been demonstrated, that hSAE cells mature into a pseudostratified epithelium (scheme in Fig. 2a) under ALI conditions^3^. In the here investigated 96-Transwell plates, the cellular composition of the epithelial layers was analyzed and compared to the 24-Transwell format. Using confocal immunofluorescence microscopy, the markers KRT5 for basal cells, SCBG1A1 for club cells, MUC5AC for goblet cells, and AcTubulin for ciliated cells were detected (Fig. 2b,c). These are the same four cell types that also make up the small airways of a human lung^3^. Furthermore, the relative proportions of the individual cell types with respect to each other were similar between 24- and 96-format (see also Supplementary Video V1 online). When a lateral view of the Transwell epithelium was reconstructed based upon a projection of the confocal scan, it was demonstrated that in both microplate formats the hSAE cells matured towards a polarized epithelium with the basal cell bodies residing on the synthetic Transwell membrane and the cilia oriented towards the upper Transwell compartment (Fig. 2d,e). For single color and overlay images of hSAE cells immunofluorescence stains see also Supplementary Figure S2 online.

The presence of functional mucus-producing cells within the 96-Transwell epithelial layer was further corroborated by staining the mucus-contained glycoproteins with Alcian Blue. Mucus secretion was scarce from a hSAE cell-based layer that had matured for 1 week under ALI conditions (Fig. 2f,g). After 4 weeks of ALI-driven differentiation, however, an extensive amount of Alcian Blue-stained mucus was detectable. Also on the mRNA level (Fig. 2h), RT-PCR analysis revealed the presence of the different cell types in both 96- and 24-Transwell formats. Mucus producing (*MUC5AC* and *MUC5B*), basal (*KRT5*) as well as ciliated cells (*FOXJ1*) could be verified on mRNA level (for detailed statistics, see Supplementary Table S1, S2 and S3 online). Additionally the comparison of hSAE cells to large airway cells revealed a significantly higher expression of surfactant protein B (*SFTPB*) (*p* < 0.0001) and significantly lower expression levels of *MUC5AC* (*p* < 0.0022), supporting the preservation of hSAE cell specific characteristics (see Supplementary Figure S3a,b online).

### Functional analysis of the hSAE cell-based epithelium in 96-Transwell format

**Figure 3:**
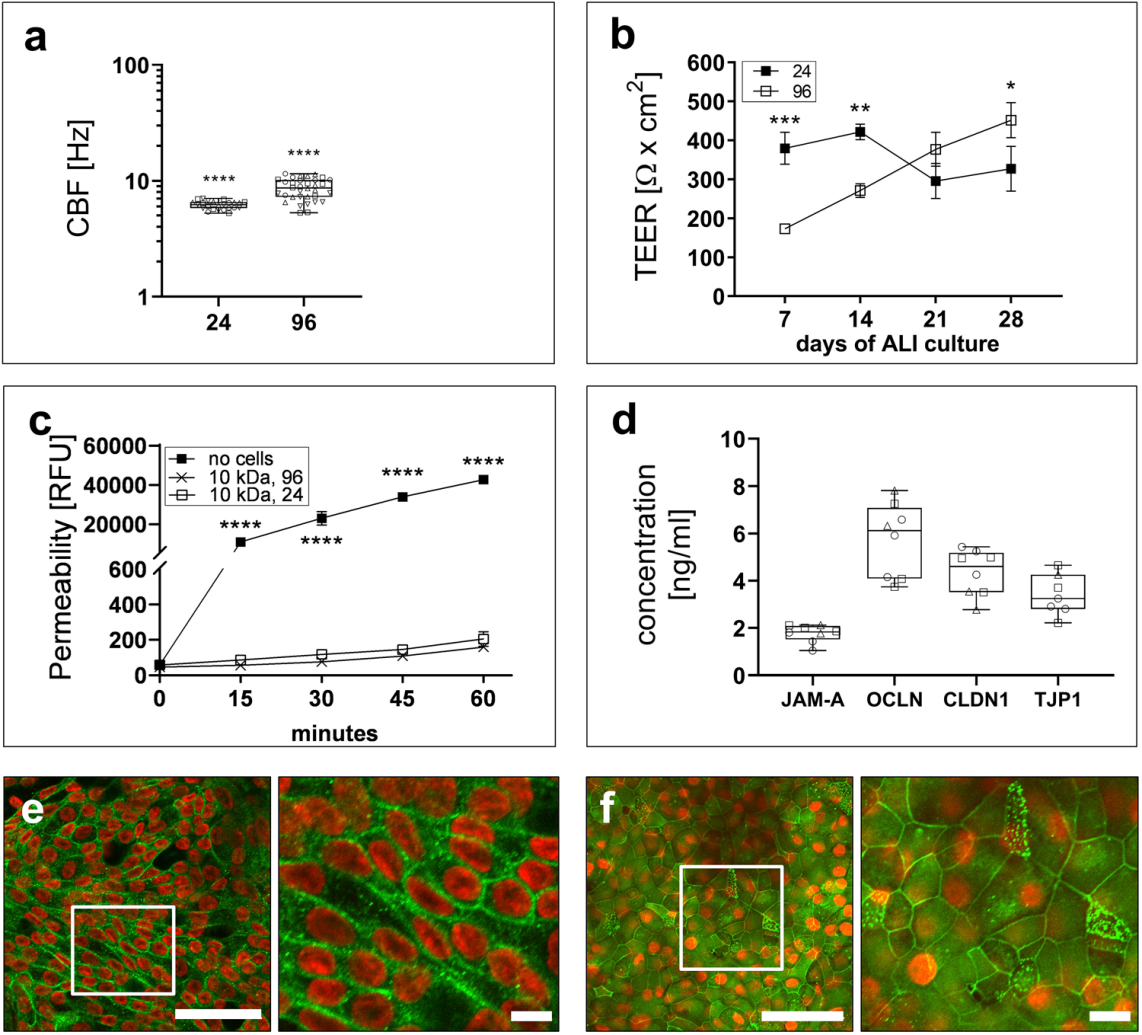
Investigation of epithelial barrier integrity in small airway epithelial cells. (**a**) Comparison of ciliary beating frequency CBF of small airway epithelium cells cultured under 24 Transwell and HTS adapted conditions (*24:* N = 4, n = 24; *96:* N = 4, n = 34). (**b**) Time course of the trans-epithelial electrical resistant (TEER) measurement, over 4 weeks of air–liquid-interface (ALI) culture, for the verification of epithelial barrier integrity under 24- and 96-Transwell conditions (*24:* N = 3; *96:* N = 48). Mean ± 95% CI. (**c**) Determination of the permeability of the epithelium (24-Transwell, 96-Transwell) via FITC-Dextran over 60 min (*24:* N = 4, n = 8; *96:* N = 4, n = 8). Mean ± 95% CI, even if not visible. (**d**) Quantification of different tight junction proteins in 96-Transwell cultured epithelia cells, classical Enzyme-Linked Immunosorbent Assay (ELISA); *JAM-A*: N = 3, n = 8; *OCLN*: N = 3, n = 8; *CLDN1*: N = 3, n = 8; *TJP1:* N = 3, n = 8. Median; range [min, max]. (**e**, **f**) Representative immunofluorescence staining in HTS adapted cells for the verification of epithelial barrier integrity based on adherence junction proteins as E-Cadherin (**e**) and tight junction proteins as TJP1 (**f**); scale bar = 50 µm/ 10 µm.

A functional small airway epithelium in vivo is characterized by synchronized beating of the cilia to transport apical mucus from the bronchioles to the proximal region of the lung. In order to validate the ciliary function of the 96-Transwell contained hSAE cell-epithelium after four weeks of maturation under ALI conditions, the ciliary beating frequency (CBF) was determined by microscopic recording combined with image analysis (see Supplementary Video V2 online). The median CBF value of 8.6; range = [5.3, 11.5] Hz for the 96-format (for detailed statistics, see Supplementary Table S2 online) was in a comparable range as the median of 6.2; range = [5.3, 7.0] Hz for the 24-format (Fig. 3a). Furthermore, the comparison to large airway cells showed significantly higher CBF values in hSAE cells, underlying the small airway origin (see Supplementary Figure S3c online).

The in vivo bronchiolar epithelium is further characterized by intercellular tight junctions that grant a protective physical barrier between the bronchial lumen and the underlying tissue. For larger Transwell formats, it has previously been shown that in vitro matured airway epithelial cells develop a barrier of high electrical resistance^13,14^. In this work, the increase of the TEER value over the first four weeks of ALI-based maturation was monitored both for 24- and 96-Transwell plates (Fig. 3b). After one week under ALI conditions, the mean TEER value of 380; 95% CI = [338.5, 420.4] Ω × cm^2^ for the 24-Transwell plate was still significantly higher than the mean TEER value of 173, 95% CI = [163, 183.1] Ω × cm^2^ for the 96-Transwell plate (*p* = 0.0003). After four weeks under ALI conditions, however, both mean TEER values of 451; 95% CI = [406.5, 496.5] Ω × cm^2^ for the 96-format and of 327, 95% CI = [269.3, 384.0] Ω × cm^2^ for the 24-format indicated a tight intercellular sealing (for detailed statistics, see Supplementary Table S4 online).

Additionally the tightness of the epithelium was validated by conducting a dextran permeability study (Fig. 3c). The flux of 10 kDa FITC-labeled dextran from the upper into the lower compartment, was significantly reduced by a four week-ALI-matured epithelial layer in comparison to the cell-free synthetic Transwell membrane. The respective reduction of the flux rate was similar between the 24- and the 96-format. The dextran flux result corroborates the above TEER-based finding that miniaturized 96-Transwell plates allow for the formation of a tight epithelium under the chosen maturation conditions (for detailed statistics, see Supplementary Table S5 online).

The epithelial integrity in vivo is maintained by tight junctions and adherens junctions^27^. A cellular lysate of the epithelium from the 96-Transwell plate, contained the junctional adhesion protein A (JAM-A), and the three tight junction proteins occludin (OCLN), claudin-1 (CLDN1) and tight junction protein-1 (TJP1; Fig. 3d). Likewise, immunofluorescence microscopy of the epithelium in the 96-Transwell plate showed the adherens junctional protein E-cadherin (Fig. 3e) and the TJP1 (Fig. 3f) after four weeks of maturation under ALI conditions. The descriptive statistics are provided in Supplementary Table S6 online.

### Detection of epithelial breakdown and subsequent changes in pro-fibrotic marker expression levels

**Figure 4:**
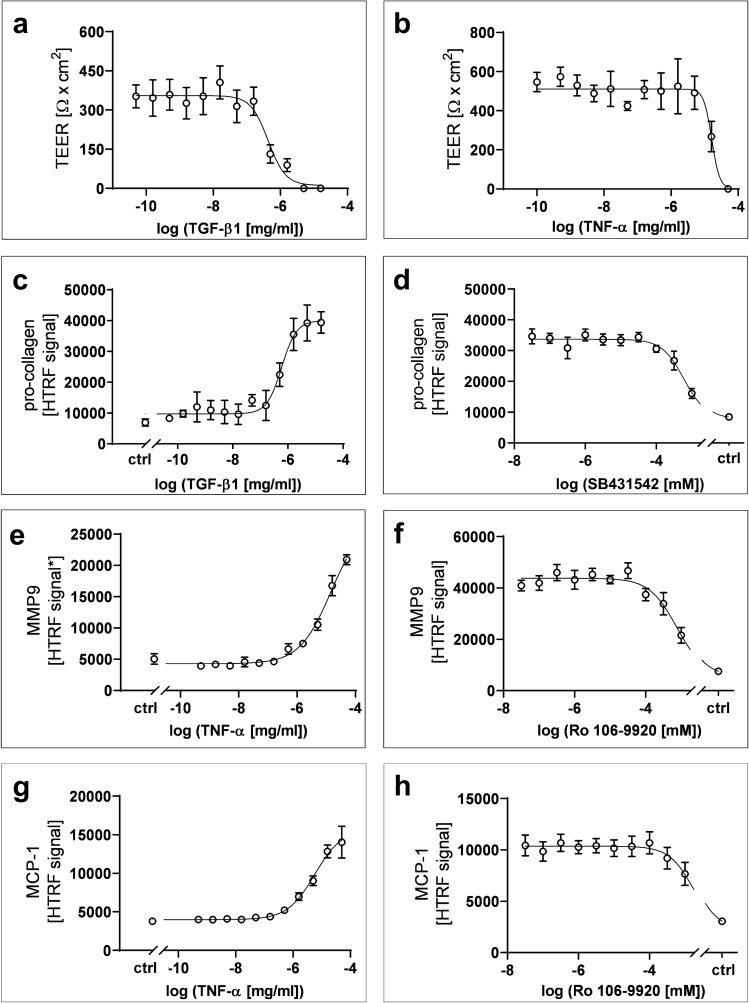
Changes in epithelial barrier function and pro-fibrotic marker expression of primary small airway epithelial cells in the presence of different cytokine concentrations and respective inhibitors (mean ± 95%CI). (**a**, **b**) Concentration dependent breakdown of barrier function and subsequent loss in TEER, in the presence of TGF-β1 (N = 10) and TNF-α (N = 7) stimulation. (**c**) Concentration dependent pro-collagen I expression in the presence of TGF-β1 stimulation (N = 3, n = 6). (**d**) Concentration dependent reduction in pro-collagen I expression in the presence of SB431542 and 1 ng/mL TGF-β1 stimulation (N = 4, n = 12). (**e**) Concentration dependent MMP9 expression in the presence of TNF-α stimulation (N = 4, n = 12). *Samples were pre-diluted 1:5 in DPBS. (**f**) Concentration dependent reduction in MMP9 expression in the presence of Ro 106-9920 and 5 ng/mL TNF-α stimulation (N = 8, n = 24). (**g**) Concentration dependent MCP-1 expression in the presence of TNF-α stimulation (N = 8, n = 24). (**h**) Concentration dependent reduction in MCP-1 expression in the presence of Ro 106-9920 and 5 ng/mL TNF-α stimulation (N = 8, n = 24).

TGF-β1 and TNF-α are key mediators of IPF disease pathogenesis. The respective cytokine challenges were replicated in vitro by administration of both TGF-β1 and TNF-α to ALI-matured hSAE cell-based epithelial layers in 96-Transwell plates (Fig. 4). In order to monitor the integrity of the epithelium, the TEER value was determined after 72 h of cytokine exposure. In dose response experiments, both TGF-β1 and TNF-α induced a concentration-dependent breakdown of the TEER-correlated epithelial barrier with mean EC50 values of 0.43; 95% CI = [0.21, 1.8] ng/mL (Fig. 4a) and 16; 95% CI = [10, 25] ng/mL (Fig. 4b), respectively. In IPF, TGF-β1 is known to induce elevated levels of collagen I deposition in vivo^20^. In agreement with the in vivo pathogenesis, TGF-β1 was found to dose-dependently increase the secretion of pro-collagen I from hSAE cells with a mean EC50 value of 0.59; 95% CI = [0.42, 0.88] ng/mL (Fig. 4c). Corroborating that this effect was mediated by a TGF-β Receptor, the selective small inhibitor SB-431542^28^ was shown to decrease the TGF-β1-driven pro-collagen I release from hSAE cells with a mean IC50 value of 0.6, 95% CI = [0.47, 0.78] µM (Fig. 4d).

TNF-α has been shown to induce the secretion of MMP9 and MCP-1 in IPF disease pathology. Reflecting this in vivo biology in the 96-Transwell plates, TNF-α induced ALI matured hSAE cells to dose-dependently increase the secretion of MMP9 with a mean EC50 value of 14; 95% CI = [9.4, 30.8] ng/mL (Fig. 4e). In vivo, NF-κB signaling is one of the central pathways downstream of TNF-α. In agreement with this signaling chain, a small molecule inhibitor of IκB ubiquitination Ro 106-9920^29^ decreased MMP9 secretion from TNF-α–stimulated hSAE cells with a mean IC50 value of 0.75, 95% CI = [0.55, 1.2] µM (Fig. 4f). Likewise, TNF-α induced hSAE cells to secrete MCP-1 with a mean EC50 value of 5.7; 95% CI = [4.2, 8.9] ng/mL (Fig. 4g). Additionally, Ro 106-9920 inhibited TNF-α driven MCP-1 release with a mean IC50 value of 1.5; 95% CI = [0.97, 4.6] µM (Fig. 4h). Donor variability was also tested to strengthen the robustness of the established assays within this work. Results are depicted in Supplementary Figure S4 online. Detailed statistics for all stimulation experiments are provide in Supplementary Table S7, S8 online.

## Discussion

This study characterized a miniaturized ALI model of primary hSAE cell epithelium with respect to its cellular composition, morphology and physiological function. Furthermore the cellular system was validated for its suitability as a drug discovery assay for respiratory disease research.

Prior to seeding the hSAE cells into Transwell microplates, a freezing step was inserted into the workflow (see supplementary Fig. S1 online). The resulting aliquoted frozen cell stocks are extremely beneficial to regularly repeated drug discovery assay cycles. Thus, typical compound optimization campaigns in medicinal chemistry produce a few dozen compounds per week, which need to be tested as soon as possible to guide the chemists with respect to the structure activity relationships within the favored compound classes. A single 96-Transwell plate may suffice to produce dose response characterization of approximately ten test compounds in singlicate measurements. Three frozen stock vials of three million pre-expanded hSAE cells each, theoretically suffice to prepare 450 wells in the 96-Transwell format with 20,000 cells per well. Considering cellular losses through the freeze–thaw cycle and the dead volumes related to liquid handling, the three vials practically translate into four 96-Transwell plates offering testing capacity for ten test compounds in dose response and in quadruplicate measurement. Depending on the actual signal window for a particular pharmacological readout, lower replicate numbers than 4 may be adequate.

In order to reduce the required manual working capacity and to increase the throughput, the handling of the 96-Transwell plates was partially automated. The usage of a Multidrop Combi Reagent Dispenser and a BioTek EL406 Washer Dispenser for the liquid handling provided fast and consistent dispensing of coating solutions, medium and cellular suspensions. In addition, the here employed 96-Transwell microplate format with relatively large access ports to the upper and lower compartment lends itself rather well to an automated procedure for medium exchange. Overall, the automated processing in the 96-Transwell format is more time-efficient and cost saving in comparison to the manual handling of 24-Transwell plates. However, the miniaturization and automation may have a negative impact on the maturation of the small airway epithelium in the microplates. For instance, the consistent aspiration of the medium during the airlift is crucial for the uniform differentiation of the primary cells across all wells of the microplate. The comparison of the cell type composition between 24- and 96-Transwell matured epithelial layers showed that both plate formats produced similar proportions of basal, club, goblet and ciliated cells.

The epithelia, differentiated in 96-Transwell plates, secreted the characteristic mucus-contained glycoproteins on the apical side. The mucus was observed to be depositioned in patches. This could be due to the heterogeneous distribution of the different cell types as well as a functional ciliary system to help clear the mucus. Indeed the CBF analysis revealed cilia clusters on the surface of the epithelium both in the 24- and the 96-format. In comparison, cell lines of the respiratory tract, such as Calu-3, had been shown to produce a uniform mucus layer on the apical surface, possibly due to the single cell type and the absence of mucociliary clearance^30,31^.

The TEER value is a broadly accepted marker for the integrity of an epithelial barrier^32^. The absolute TEER value for the matured hSAE cell-based epithelium in the 96-Transwell microplates was similar to those previously obtained in a larger Transwell format^13^. This corroborated the homogeneous establishment of intercellular tight junctions in the miniaturized format. Disadvantageously, the TEER measurement is rather sensitive to parameters such as temperature or the geometric arrangement of the electrodes with respect to the epithelial layer. Thus, standard TEER measurements using single chopstick electrode pair for sequential measurements across a Transwell microplate typically suffer temperature effects associated with chilling down of the microplate after taking out from a cell incubator. In this work, a novel TEER device was developed that carried out the ohmic resistance measurements for all 96 Transwells of a plate in parallel.

This device also addressed the second issue of the manually operated single chopstick electrode pair, i.e. that for each measurement the distance between the electrodes and the membrane layer may slightly vary. In contrast, the 96-electrode pair array contained in the novel TEER device granted a fixed and reproducible positioning of the electrodes with respect to the Transwell synthetic membranes. Whereas manual handling of chopstick electrodes bears the danger of physically damaging the epithelial layer, the electrodes in the 96-well array have a defined depth of penetration into the upper compartments of the Transwell plates leaving a defined distance to the epithelium. In addition, the electrodes of the 96-well TEER device are automatically washed with both ethanol and sterile water. In consequence, there is less danger of cross-well contaminations than with chopstick electrodes and a manual washing process prone to human error. Hence, the 96-well TEER device combines the advantages of reproducibility, accuracy, ease-of-operation, throughput and decreased risk of contamination.

As an orthogonal technique to investigate the functional integrity of epithelial barrier, FITC dextran (paracellular) flux assays were performed. While often the effects of barrier disruption produce comparable effects on the TEER value and the flux measurement, there are for instance some pharmacological agents such as dexamethasone that alter TEER, but not paracellular transport of molecules^33^. In these cases, the flux measurements more closely reflect the functional barrier integrity. In this work, the epithelial layers matured in the 24- and 96-Transwell format displayed a comparable reduction of paracellular flux, an observation which paralleled the similar TEER values between both plate types.

Molecularly, the barrier between the subepithelial tissue and the airway lumen is primarily established by the tight junctions and the adherens junctions^9^. Tight junctions are formed as apical rings around the epithelial cells. They are composed of transmembrane proteins such as occludin, claudin-1, and junctional adhesion protein A as well as scaffolding proteins such as tight junction protein-1. Adherence Junctions consist of E-cadherin and multiple catenins, which connect to the actin cytoskeleton via anchoring proteins (e.g., cortactin and cingulin). Using ELISA, the presence of the above mentioned tight junction proteins in the lysate of 96-Transwell ALI-matured hSAE cells was demonstrated. In immunofluorescence imaging, E-cadherin and tight junction protein-1 were found at the contact points between cells across the epithelium, consistent with the formation of a polarized epithelial barrier.

Exaggerated TGF-β1 signaling is also strongly implicated in numerous fibrotic diseases^11,19,34^. For instance, when exposed to TGF-β1, the TEER value across epithelial layers of both primary rat alveolar and primary human bronchioepithelial cells had previously been shown to be significantly decreased^35^. Similarly in this work, the electric resistance of an ALI-matured layer of hSAE cells declined after stimulation with TGF-β1. The mean EC50 value for this TGF-β1-induced TEER reduction of 0.43, 95% CI = [0.21, 1.8] ng/mL was in the same dose range as the previously observed effective concentrations of TGF-β1 in the above described experiments on primary rat alveolar and human bronchioepithelial cells^35^.

TGF-β1 is a key driver of collagen accumulation in fibrotic disease^34^. In agreement with this fact, TGF-β1 induced the secretion of pro-collagen from hSAE cells at a mean EC50 value of 0.59, 95% CI = [0.42, 0.88] ng/mL. This EC50 value also corresponded well with the above described TGF-β1-based modulation of the TEER value. Likewise, 10 ng/mL of TGF-β1 had previously been demonstrated to increase the secretion of collagen from alveolar epithelial cells^36^. SB431542, a potent and selective inhibitor of the TGF-β Superfamily Type I Activin Receptor-Like Kinase (ALK) Receptors ALK4, ALK5, and ALK7^28^^,^ prevented the TGF-β1 induced secretion of pro-collagen from the hSAE cells with a mean IC50 value of 0.6, 95% CI = [0.47, 0.78] µM. This value corresponded well with the previously measured IC50 values of 1 µM, 0.75 µM, and 2 µM for ALK4, ALK5, and ALK7, respectively, in a SMAD2/4-based reporter gene assay^28^.

Like TGF-β1, TNF-α is an important mediator of lung fibrotic pathology^21^. Increased levels of TNF-α have been found in the lungs of IPF patients and in animal models for pulmonary fibrosis. Previously, 10 ng/mL of TNF-α had been observed to lower the TEER value across a confluent layer of ALI-conditioned A549 cells^37^. Similar to this dose effect, TNF-α reduced the electric resistance across a layer of ALI-cultured hSAE cells with a mean EC50 value of 16, 95% CI = [10, 25] ng/mL. In human airway epithelial cells obtained by bronchial brushings, 10 ng/mL of TNF-α had induced a markedly upregulated secretion of MMP9 and MCP-1^38^. Correspondingly, TNF-α induced the release of MMP9 and MCP-1 from the hSAE cells with mean EC50 values of 14, 95% CI = [9.4, 30.8] ng/mL and 5.7; 95% CI = [4.2, 8.9] ng/mL, respectively. In further agreement with these data, Propst et al.^39^ had found TNF-α to stimulate the release of MCP-1 from immortalized human airway epithelial 9HTEo^-^ cells at a concentration of 1 ng/mL.

TNF-α had earlier been recognized to stimulate nuclear factor kappa B (NF-κB)-mediated signaling^37^. In accordance with the involvement of this signaling chain in the TNF-α-driven activation of airway epithelium, the small molecule inhibitor of IκB ubiquitination Ro 106-9920^29^ decreased the secretion of MMP9 and MCP-1 from TNF-α–stimulated hSAE cells with mean IC50 values of 0.75, 95% CI = [0.55, 1.2] µM and 1.5; 95% CI = [0.97, 4.6] µM, respectively. Furthermore, Swinney et al.^29^ had observed that Ro 106-9920 inhibited the TNF-α–induced degradation of IκB in Monomac 6 cells with an IC50 value of approximately 1 µM.

In summary, the application of a 96-Transwell microplate system to hSAE cells in ALI culture has been characterized. The hSAE cells in this miniaturized system displayed similar features compared to the previously established 24-Transwell and larger formats with respect to (i) cellular differentiation into a pseudostratified, polarized epithelium, (ii) ciliary beating, (iii) mucus production, (iv) establishment of intercellular tight junctions, and (v) responsiveness to pathophysiological stimuli as exemplified by TGF-β1 and TNF-α. In addition, the applicability to small molecule pharmacological investigations was demonstrated. The established system provides a unique opportunity to study IPF in vitro and to analyze the effects of test compounds on airway epithelium. Further exploration of the cellular model in regard to disease relevant stimulation is warranted to better understand the pathology of IPF and identify new innovative solutions on how to treat the disease.

## Methods

### Primary cell culture: cryopreservation, expansion and Air–Liquid Interface cell culture

hSAE cells, isolated from the distal portion of the human lung (1 mm bronchiole area), were purchased from Lonza (cat. CC-2547S, Lonza, Basel, Switzerland). As stated by Lonza, the cells were isolated from donated human tissue after obtaining permission for their use in research applications by informed consent or legal authorization (see Supplementary Methods online).

Cryopreserved cells (healthy donor #501937, seeding efficiency: 85%; doubling time: 30 h; viability: 93%; ≤ 500,000 cells per vial) were cultured and differentiated according to manufacturer’s instructions. Briefly, cells were expanded in a T175 Nunclon Delta surface-treated EasYFlask (cat. 178883, Thermo Fisher Scientific, Waltham, MA) using the “expansion medium” that consisted of Pneumacult-Ex Plus medium (cat. 05040, Stemcell Technologies, Vancouver, Canada), supplemented with 0.1% (v/v) hydrocortisone (cat. 07925, Stemcell Technologies) and 1% (v/v) Penicillin/Streptomycin (cat. 15140-122, Thermo Fisher Scientific). hSAE cells were cultured at 37 °C and under 5% CO_2_ conditions, with a medium change every other day.

After reaching ~ 70% confluency, the cells were passaged into 3 × T175 Nunclon Delta surface-treated EasYFlasks using an Animal Component-Free Cell Dissociation Kit (cat. 5426, Stemcell Technologies) for 6 min at 37 °C. The detached cells were centrifuged at 200 g for 5 min and re-suspended in the “expansion medium”. Cells were cultured for additional 3–4 days in the “expansion medium”, with medium change every other day. Subsequently, the cells were cryopreserved as frozen stocks at passage 2 in CryoStor CS10 (cat. 7931, Stemcell Technologies) for long-term storage.

Prior to cell seeding, filters of either 96-Transwell plates (cat. CLS7369, Corning, Corning, NY) or 24-Transwell plates (cat. 3470, Corning) were pre-coated with 50 µL or 100 µL, resp., of 0.33 mg/mL of rat-tail collagen I (cat. 354236, Corning) for 45 min at 37 °C and 5% CO_2_.

From frozen stocks, the cells were diluted in “expansion medium”. Then 20,000 cells/well in 50 µL/well were dispensed into the collagen-coated 96-Transwell plates using a Multidrop Combi reagent dispenser (cat. 5840300, Thermo Fisher Scientific), and 30,000 cells/well in 100 µL/well were manually pipetted into the collagen-coated 24-Transwell plates. In both plate types, cells were cultured in “expansion medium” under submerged conditions for 2 days. For the 96-Transwell plates, the medium change in apical and basal compartments was done using a BioTek EL406 Washer Dispenser (BioTek Instruments Inc., Winooski, VT). For the 24-Transwell plates, the respective medium change in apical and basal compartments was carried out manually. In both plate types, cells were cultured in “expansion medium” under submerged conditions for another 2 days.

The air–liquid interface (ALI) condition was initiated 4 days post-seeding, by the complete aspiration of the medium in the apical compartment, and by replacement of the “expansion medium” in the basal compartment by the “ALI medium”, which consisted of PneumaCult-ALI-S basal medium (cat. 5099, Stemcell Technologies) containing 10% (v/v) PneumaCult-ALI-S 10 × Supplement (cat. 5003, Stemcell Technologies), 1% (v/v) PneumaCult-ALI Maintenance Supplement (cat. 5006, Stemcell Technologies), 0.2% (v/v) heparin solution (cat. 7980; 0.2% stock concentration, Stemcell Technologies), 0.5% (v/v) hydrocortisone (cat. 07925, Stemcell Technologies) and 1% (v/v) Penicillin/Streptomycin (cat. 15140-122, Thermo Fisher Scientific).

During maturation of cells, the “ALI medium” was replaced three times a week. For the 96-Transwell plates this medium change was carried out by using a BioTek EL406 Washer Dispenser integrated into the below described automated cell culture system. The differentiation in 24-Transwell plates followed the same schedule, but it was done manually. hSAE cells were differentiated under ALI conditions for 4 weeks, after which mucus production and ciliary beating were observable.

### Measurement of epithelial barrier integrity

Transepithelial electrical resistance (TEER) across the epithelial layer in the Transwell plates was measured weekly. Since the dimensions of the conventional STX2 electrode was too big (diameter of 4 mm) to enter the basolateral access port of the 96 Transwell tray (~ 2.5 mm), a custom-built, automated 96-electrode device was employed for the 96-Transwell plates. For a detailed description of the novel device see Supplementary Methods online. Prior to the TEER measurements, cells were manually washed once with pre-warmed 1 × DPBS (cat. 14190144, Thermo Fisher Scientific) to remove excessive mucus. For the 24-Transwell plates, the TEER measurements were carried out using a STX-2 chopstick electrode attached to an epithelial voltohmmeter (EVOM, World Precision Instruments, USA). Any excessive mucus was as well removed by washing the cells on the apical side once with 1 × DPBS. Subsequently 120 µL/well or 250 µL/well of pre-warmed “ALI medium” was added to the apical chambers of the 96- or 24-Transwell plates, resp., to allow for the electrical measurement. The removal time from the incubator of the plates were comparable in both formats. Raw data were corrected by subtracting the electrical resistance as measured across a cell-free Transwell insert. The final TEER values were obtained by multiplication with the area/well of synthetic Transwell membrane.

Additionally, paracellular flux studies were performed to evaluate barrier integrity and proper tight junction formation. Therefore, a FITC-Dextran permeability assay was conducted with hSAE cells after 4 weeks of ALI culture. Briefly, after aspiration of the medium from the basolateral chambers and a 1 × DPBS washing step, 150 µL/well or 500 µL/well of pre-warmed RPMI w/o phenolred (cat. 11835-063, Gibco, Waltham, MA) was added to the basolateral chambers of the 96- or 24-Transwell plates, respectively. Next, 60 μL or 200 µL of a 5 mg/mL FITC-Dextran solution (cat. FD10S-1G, Sigma-Aldrich, St. Louis, MO) was added to the apical chambers of the 96- or 24-Transwell plates, respectively. Then the plates were incubated at 37 °C and 5% CO_2_. At the indicated time points, 10 μL-samples were taken from the basolateral chamber and transferred into a black 384-well plate (cat. 781076, Greiner Bio-One, Frickenhausen, Germany). FITC fluorescence reading was measured using a SpectraMax M5 (cat. M5, Molecular Devices, San Jose, CA) plate reader with excitation and emission wavelength settings of 490 and 520 nm, respectively.

### Immunofluorescence of markers for hSAE cell types and tight junctions

Marker proteins of the four different hSAE cell types and of the tight junctions were visualized by immunolabeling and confocal microscopy. The hSAE were sampled for immunofluorescence staining four weeks post-ALI. Subsequently cells were washed once with 1 × DPBS, then fixed with 4% (v/v) paraformaldehyde solution (cat. 252549-500ml, Sigma-Aldrich) for 15 min at room temperature. Subsequently, the inserts were washed three times with 1 × DPBS, then permeabilized with 0.3% (v/v) Triton X-100 (cat. T8787-100 ml, Sigma- Aldrich) in 5% (w/v) Bovine Serum Albumin (BSA) (cat. A3059-100G, Sigma-Aldrich) in 1 × DPBS for 60 min at room temperature. After three additional washing steps with 1 × DPBS, the fixed cells were incubated at 4 °C over night with the indicated primary antibodies (see Table 1 of antibodies and respective dilution factors) and 2.5 µg/mL Hoechst 33342 (cat. H3570, Thermo Fisher Scientific) diluted in 0.5% (v/v) BSA in 1 × DPBS. The next day, the inserts were washed three times with 1 × DPBS and incubated in the dark with species-specific secondary Alexa Fluor antibodies (see Table 1) diluted in 0.5% (v/v) BSA in 1 × DPBS for 1 h at room temperature. This step was skipped for the already pre-labeled primary antibodies. Finally, the inserts were washed three times with 1 × DPBS and cut from the plastic support, for subsequent mounting on a microscope slide using the ProLong Diamond Antifade Mountant (cat. P36961, Thermo Fisher Scientific). Imaging was performed using a LSM710 laser confocal microscope (Carl Zeiss Microscopy, Jena, Germany). For technical settings of the instrument see Supplementary Table S9 online.

**Table 1:** List of primary and secondary antibodies used in this study.

Antibody	Dilution	Cat. No	Vendor
MUC5AC (mouse IgG1k) CF488 conjugated	1:50	MA1-38223	Invitrogen
KRT5 (rabbit IgG) Alexa Fluor 647 conjugated	1:100	ab193895	abcam
SCGB1A1 (rat IgG1)	1:50	MAB4218-SP	Novus biologicals
Ac-Tubulin (mouse IgG2b) CF405 conjugated	1:1,000	12152S	Cell signaling
E-Cadherin (rabbit IgG)	1:200	3195S	Cell signaling
ZO-1 (goat IgG)	1:500	PA5-19090	Invitrogen
Goat anti rat IgG, Alexa Fluor 568	1:500	A-11077	Invitrogen
Goat anti mouse IgG Alexa Fluor 488 conjugated	1:500	A-11029	Invitrogen
Goat anti mouse IgG1 Alexa Fluor 568 conjugated	1:500	A-21124	Invitrogen
Goat anti rabbit IgG Alexa Fluor 488 conjugated	1:500	A-11034	Invitrogen
Rabbit anti goat IgG Alexa Fluor 546 conjugated	1:500	A-21085	Invitrogen

### Quantification of Tight Junction Proteins

Tight junction and adherence function protein concentrations in cell culture lysates were determined using the Sandwich-ELISA principle. To detect total human occludin (OCLN; cat. E-EL-H1073, Elabscience, Houston, TX), claudin-1 (CLDN1; cat. E-EL-H0745, Elabscience), tight junction protein 1 (TJP1; cat. E-EL-H1516, Elabscience) and junctional adhesion molecule A (JAM-A; cat. KOA0813, Rockland, Limerick, PA), the respective kits were used according to the manufacturer’s instructions. Quantification of protein concentrations was determined by the comparison to an internal protein standard and the respective data.

### Gene expression profiles

For gene expression analysis, cellular lysis in RLT Plus buffer (cat. 79216, Qiagen, Venlo, Netherlands) and RNA isolation were performed using the RNeasy Plus Mini Kit (cat. 74134, Qiagen) according to the manufacturer’s instructions. Reverse transcription reaction was accomplished using the Applied Biosystems High-Capacity cDNA Reverse Transcription Kit (cat. 4368814, Thermo Fisher Scientific) according to the manufacturer’s instructions. For comparability, the cDNA concentration in each sample was adjusted to 10 µg/mL, and 4 µL/sample were added to a final reaction volume of 20 µL containing the TaqMan Fast Advanced Master Mix (cat. 4444556, Thermo Fisher Scientific) and the respective intron-spanning Applied Biosystems TaqMan Gene Expression Assay (cat. 4351370, FAM Dye, Thermo Fisher Scientific), as listed in Table 2. Gene expression levels of single genes were normalized to the reference gene encoding the RNA Polymerase II Subunit A (*POLR2A*). The derived 2^(−Δct)^ values of the differentiated samples were recalculated to 2^(−ΔΔct)^ values in reference to the corresponding 2^(−Δct)^ values of the non-differentiated basal cells.

**Table 2:** List of TaqMan gene expression assays used within this study.

Target	Assay ID
*KRT5*	Hs00361185_m1
*MUC5AC*	Hs01365616_m1
*MUC5B*	Hs00861595_m1
*FOXJ1*	Hs00230964_m1
*POLR2A*	Hs00172187_m1

### Mucus production

For the analysis of mucus production by the hSAE cells, the presence of glycoproteins was determined after one week and four weeks of ALI culture. For visualization of the mucus on top of the cells, the glycoproteins were stained with Alcian Blue as previously described^30^. Shortly, cells were fixed with 4% (v/v) paraformaldehyde for 15 min at room temperature. After three washing steps, fixed cells were covered with a solution of 1% (w/v) Alcian Blue in 3% (v/v) acetic acid/ dH_2_O pH 2.5 (cat. B8438-250ML, Merck KGaA, Darmstadt, Germany) and incubated for 15 min at room temperature. The inserts were cut from the plastic support and transferred into 1 × DPBS to remove excessive stain. Subsequently, cells were mounted onto microscope slides using the ProLong Diamond Antifade Mountant. Imaging was performed using an inverse Axiovert.A1 microscope equipped with an Axiocam 305 color camera (Carl Zeiss Microscopy, Jena, Germany).

### High speed video analysis of ciliary function

The ciliary beating of the respective hSAE cells was investigated after four weeks of ALI culture based on a recently published method^40^. Prior to cell imaging, cells were washed at the apical site three times with pre-warmed 1 × DPBS to remove mucus. Subsequently Transwell plates were placed within an environmental controlled chamber heated to 37 °C (Solent Scientific, U.K.). Ciliary beating frequency (CBF) was recorded using a 32 × objective of an Axiovert 25 microscope (Zeiss, Oberkochen, Germany) and an acA 1,300–200 µm black and white USB-3.0 high speed camera (Basler, Ahrensburg, Germany). Four randomly selected regions of a single Transwell insert were imaged. Ciliary movement was recorded at 100 frames per second for a total of 6 s per region. Applications for image capture and analysis were developed using the Halcon 13.0.2 machine vision software toolbox (MVTec Software, Munich, Germany). Visualization of the image stacks was performed using the Analyze software (AnalyzeDirect, Overland Park, KS). A grey value time course was calculated for each pixel over 512 frames per region. The mean ciliary beating frequency of a region was determined by calculating the average frequency for the cyclic changes of the grey scale. The area covered by motile cilia was determined by quantifying the percentage of pixels with a measurable beating frequency within a region.

### Stimulation of cells

To investigate the physiological epithelial breakdown of the hSAE cells by a certain stimulus, the cells were stimulated with the indicated concentration of either TGFβ-1 (cat. 240-B, R&D Systems, Minneapolis, MN) or TNF-α (cat. 210-TA-020, R&D Systems) after four weeks of ALI culture. For this purpose, stimulants and vehicle negative controls were diluted in “ALI medium” (see above) and added to empty receiver plates (cat. 3382, Corning). Subsequently, Transwell inserts from the cell plates were transferred to the receiver plates. Stimulation was carried out for 72 h at 37 °C and 5% CO_2_, while vehicle-treated cells served as controls.

In order to test whether SB431542 (cat. 1614, Tocris Bioscience, Bristol, UK), a known inhibitor of some TGF-β receptors, decreased the TGF-β1-driven secretion of pro-collagen in hSAE cells, the compound was applied together with 5 ng/mL TGF-β1. Compound and TGF-β1 were diluted in “ALI medium” and administered for 72 h at 37 °C and 5% CO_2_, while vehicle-treated cells served as controls. In order to test whether Ro 106-9920 (cat. 1778, Tocris Bioscience), a known inhibitor of NF-kB activation, decreased the TNF-α-driven secretion of MMP9 or MCP-1 in hSAE cells, the compound was applied together with 5 ng/mL TNF-α. Compound and TNF-α were diluted in “ALI medium” and administered for 72 h at 37 °C and 5% CO_2_, while vehicle-treated cells served as controls.

After compound and/or TGF-β1/TNF-α treatment, the medium from the basal compartment was collected and frozen at − 20 °C until further analysis using time resolved fluorescence resonance energy transfer (TR-FRET), as described below.

### TR-FRET measurement of pro-fibrotic marker proteins

TR-FRET was used to measure the pro-fibrotic markers, pro-collagen, MCP-1 and MMP9 in basal cell culture supernatants after stimulation of the hSAE cells as described above. The assay was performed according to the manufacturer’s instructions (CisBio) in 384-well microplates (cat. 781075, Greiner Bio-One, Frickenhausen, Germany) with a total assay volume of 15 µl/well. In short, sandwich pairs of Eu-cryptate- and d2-conjugated anti-pro-collagen/ anti-MCP-1/ anti-MMP9 antibodies (cat. 63ADK014PEH, cat. 62HCCL2PEH, 62MMP9PEH, Cisbio, Codolet, France) were diluted with PPI Buffer (cat. 61DB9RDF, Cisbio, Codolet, France) as indicated in the respective manual. 5 µl/well of these sandwich antibody solutions were mixed with 10 µl/well of the above taken basal “supernatants”, which had been 1:5 diluted in “ALI medium” for the MCP-1 measurement. Some wells of the microplate received pro-collagen (cat. 6220-CL-020, R&D Systems), MCP-1 (cat. 300-04, PeproTech, Rocky Hill, NJ) or MMP9 (cat. 911-MP-010, R&D Systems) standard solutions instead of supernatants, so that the absolute concentrations of pro-collagen, MCP-1 and MMP9 in the supernatants could be determined. The three recombinant proteins were used, to ensure, that the actual measurements were in the dynamic range of the standard curve. Plates were incubated for 3 h at room temperature (pro-collagen) or for 24 h at 4 °C (MCP-1, MMP9), and were measured using an EnVision-Reader (excitation: 320 nm; emission: 615 nm and 665 nm) from PerkinElmer. HTRF ratio values were calculated as follows: HTRF ratio = 10,000 × emission @665 nm/emission @615 nm. The measured HTRF ratios were “transformed” into absolute protein concentrations of MCP-1 and MMP9, based upon a comparison with the HTRF ratios of the standard solutions.

### Data analysis

All data presented in this work are depicted as mean with error bars representing the 95% CI (confidence interval) of at least three independent experiments. Biological replicates (N) are defined as individual micro wells and measured independently. Technical replicates (n) were generated out of the biological replicates, respectively. In the boxplots, the horizontal line indicates the median, the box the interquartile range (25th to 75th percentiles) and the bars represent the range [min, max]. Solitary exception are qPCR, CBF and ELISA data, which are shown as box blots with median and bars representing the minimum to maximum whiskers of at least three independent experiments. 2^(−ΔΔct)^ MUC5AC and 2^(−ΔΔct)^ MUC5B qPCR data were statistically compared to a value of 1 using one-sample t-tests for each group (24 and 96 Transwell respectively). Additionally, CBF and 2^(−Δct)^ FOXJ1 qPCR data were statistically compared to a value of 0 using one-sample t-tests for each group (24 and 96 Transwell respectively). Furthermore, 2^(−Δct)^ KRT5 qPCR data were statistically compared to 2^(−Δct)^ of basal cells using the Mann–Whitney U-test. Experiments containing three or more conditions were assessed by two-way ANOVA, followed by the Fisher’s LSD test. Non-linear fits were based on the response vs. log (inhibitor) curves and were further used for the calculation of the half maximal effective concentration (EC50). Generally the nominal alpha level was set to 5% for statistical analysis in an exploratory manner and p-values of the corresponding F-statistics are presented (*p ˂ 0.05, **p < 0.01, ***p < 0.001 and ****p < 0.0001). Data input, processing, management and analyses were conducted using GraphPad Prism 8.0 (GraphPad Software).

## Supplementary information

Supplementary information

Supplementary Video S1

Supplementary Video S2
