# Supplementary material for: Development of a miniaturized 96-Transwell air–liquid interface human small airway epithelial model
Source: Sci Rep. 2020 Aug 3;10:13022. doi: 10.1038/s41598-020-69948-2 (PMC7400554; doi:10.1038/s41598-020-69948-2)
Supplement: Supplementary file 1 — Supplementary information [file 41598_2020_69948_MOESM1_ESM.pdf]

Supplementary Information

Development of a miniaturized 96-Transwell air-liquid interface human small airway epithelial model

Teresa Bluhmki[1]*, Sarah Bitzer[1], Julia Anna Gindele[2], Eva Schruf[2], Tobias Kiechle[3], Megan Webster[2], Jürgen Schymeinsky[3], Robert Ries[1], Florian Gantner[4], Daniel Bischoff[1], James Garnett[2], Ralf Heilker[1]

Departments of [1]Drug Discovery Sciences, [2]Immunology & Respiratory Diseases Research, [3]Cardiometabolic Diseases Research, Boehringer Ingelheim Pharma GmbH & Co. KG; [4]Department of Translational Medicine and Clinical Pharmacology, C. H. Boehringer Sohn AG & Co. KG, 88397 Biberach an der Riss, Germany.

* Corresponding author

teresa.bluhmki@boehringer-ingelheim.com

Supplementary Method

Details of the customized constructed transepithelial electrical resistance (TEER) device

TEER values have proven to be simple and rapid indicators of cellular barrier integrity and can be collected in real time without cell damage[1]. Therefore, this non-invasive technique has been miniaturized to suit well for the continuous monitoring of the barrier function of hSAECs during their various stages of growth and differentiation in 96 Transwell plates.

In general, TEER is the measurement of electrical resistance across any cellular layer and is a very sensitive and reliable method. The electrode components in the here implemented novel TEER device are constructed based on literature[1] and in following the structure of the classical STX2 “chopstick” electrodes.

Primarily, the collection of TEER values works based on the Ohm’s Law Method. For electrical measurements, two individual electrode pairs are used, with 2 electrodes place in the upper compartment and the other two in the lower compartment, separated by the cellular layer. One pair is made out of silver for applying the alternating current voltage throughout the cell layer. An alternating current square wave at a frequency of 12.5 Hz is applied. An additional silver electrode pair for the measurement of the resulting potential differences is placed into the individual two compartments respectively. Subsequently the ohmic resistance is calculated based on Ohm’s law as the ration of the voltage and current. The novel TEER device has a measurement range of 1-10000 Ω with a resolution of 1 Ω.

The calculation of the actual TEER values include measuring the blank resistance of the semipermeable membrane only (without cells), just medium/ buffer (was determined at the setup of the device). Determination of the resistance across the cell layer in 96 Transwell plates is performed simultaneously and three times in a row. The cell-specific resistance is subsequently calculated as the actual tissue resistance subtracted by the blank resistance. The average out of the three independent measurements per Transwell are displayed in the implemented data acquisition tool. Typically TEER values are reported in units of $\Omega \times cm^2$ to incorporate the growth area.

In general, TEER readings are highly dependent on the positioning of the electrodes, temperature and the current density generated by the electrodes across the cell layer. The here introduced self-constructed TEER device circumvents these difficulties due to the speed of each measurement and the reproducible placement of the electrodes within the Transwells. Thus, this novel technique offers the possibility to enable TEER measurements on an increased throughput.

Details of the cell isolation and characterization process of primary human small airway epithelial cells

Human small airway epithelial cells were isolated through an enzymatic digestion protocol from normal cadaveric lung tissue samples located at the lower end of either left or right lung (in the 1mm bronchiole area). Isolated cells were expanded in standard submerged culture, passaged once and subsequently cryopreserved at 500,000 cells per vial. All lots are tested for cytokeratin 19, desmoplactin and occludin in early and later stages of the differentiation process. The within this studies used donor (CC-2547S, Batch No: 0000501937) was also further characterized by mucin expression starting at day 20. TEER was detectable by day 7-10 and beta-Tubulin (Cilia) was determined by day 20. Whereas ZO-1 staining was already measurable on day 9.

Supplementary Figures

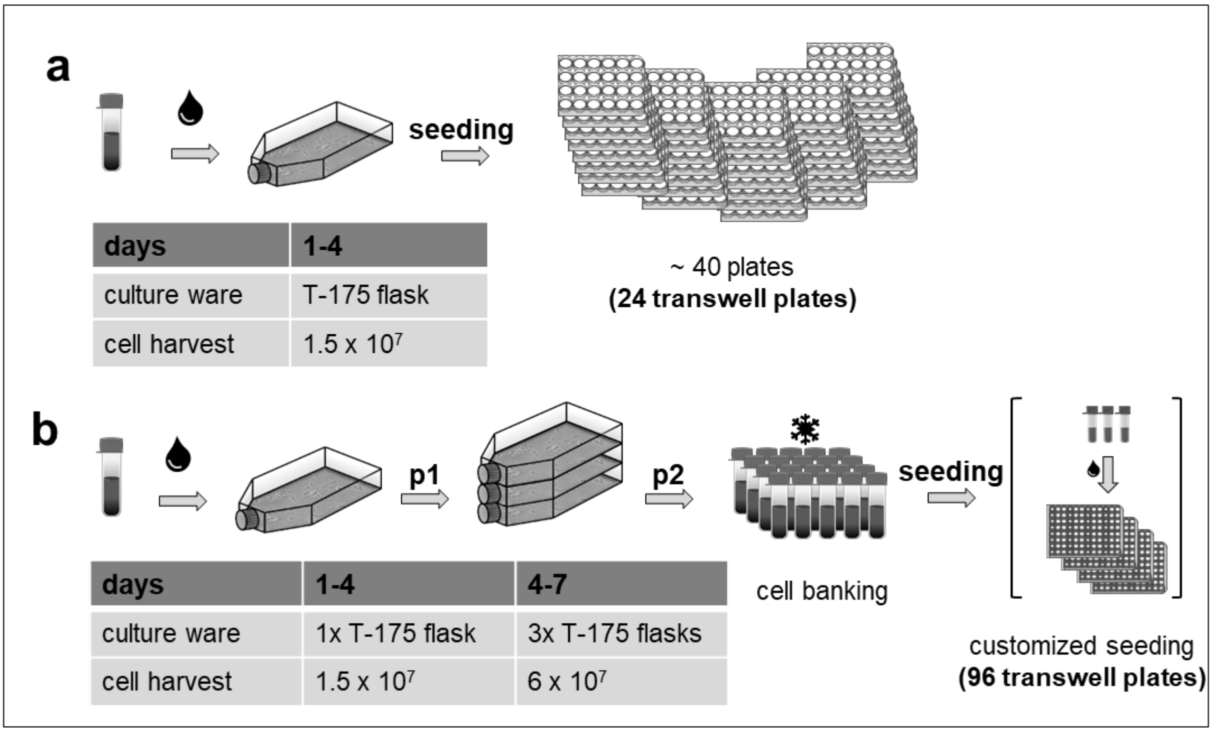

Figure S1. Upscaling and higher throughput adaptation of hSAE cell pre-culture. (**a**) 24-Transwell culture and seeding procedure of hSAE cells. The number of cells increased from 1×10^{6} to 1.5×10^{7} cells over four days of expansion culture. (**b**) Scheme of hSAE cell expansion. During large-scale cell expansion and an additional passaging step, the number of hSAE cells increased over seven days from 1×10^{6} to 6×10^{7} cells, covering the scope of an HTS campaign. The expanded hSAE cells were frozen at this stage in aliquots of ~3×10^{6} cells per vial for subsequent thawing and seeding in 96-Transwell plates. This freezing step is convenient for typical drug discovery applications, where weekly compound optimization cycles may only require a small fraction of the totally harvested 60 million cells. While the standard protocol produced twenty 24-Transwell plates representing 480 data points, the here established protocol provided sufficient cell numbers for eight profiling batches of four 96-Transwell plates each, corresponding to a total number of 3072 data points.

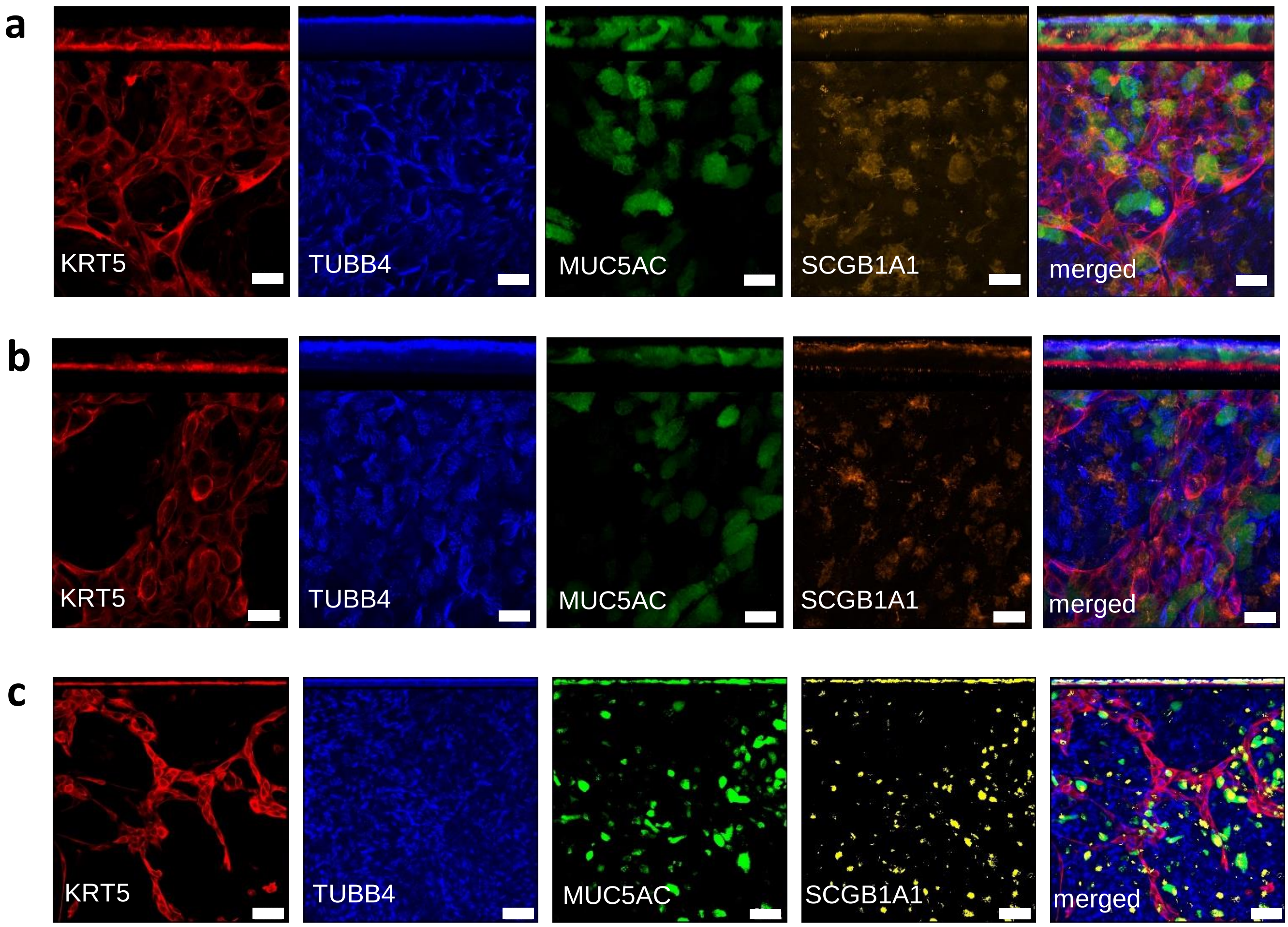

Figure S2. **Single color and overlay images of hSAE cells immunofluorescence stains. (a+b)** Confocal images (XY 3D) of the four different cell types present in small airway epithelial cell culture, cultured at the air−liquid interface after four weeks under either 24-Transwell (a) or 96-Transwell (b) conditions. KRT5 for basal cells (red), AcTubulin for ciliated cells (blue), MUC5AC for goblet cells (green) and SCGB1A1 for club cells (yellow). Scale bar = 20µm. **(c)** Images shown are representative the HTS adapted 96-well culture at a lower magnification (20x). Scale bar = 50 µm.

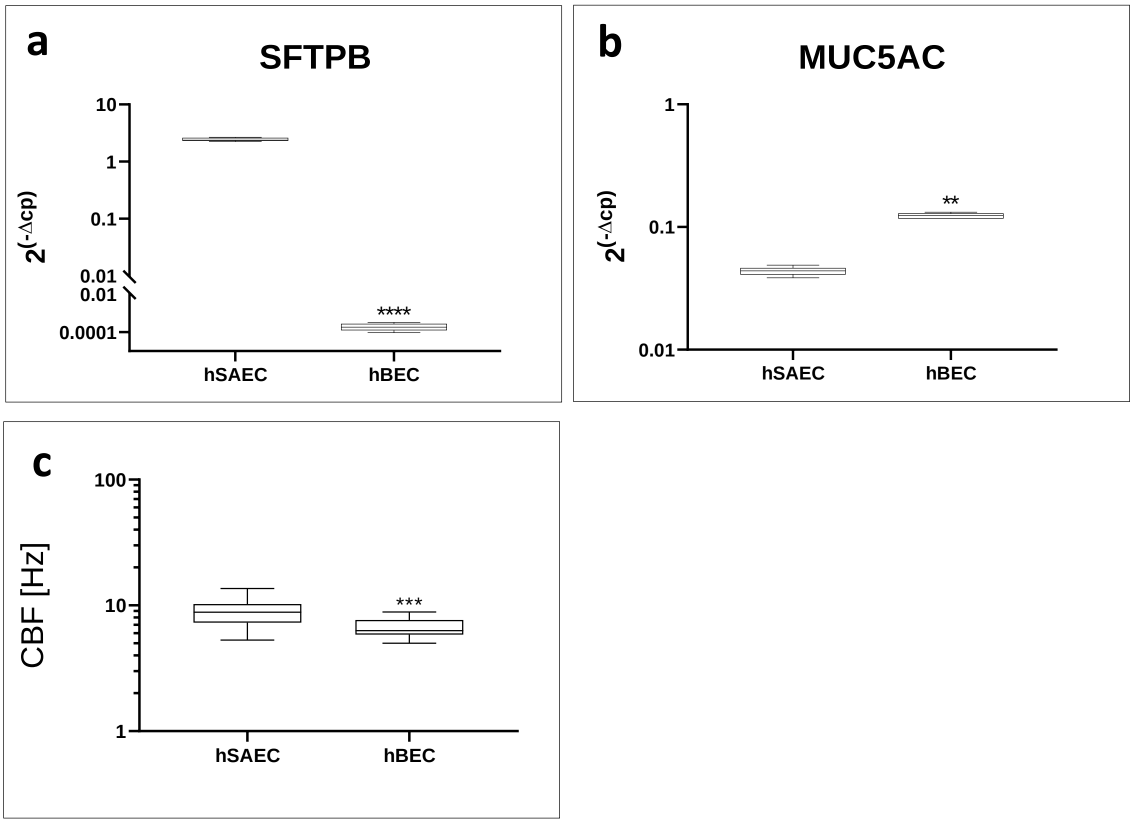

Figure S3. Comparison of small airway epithelial cell (hSAEC) and large airway (hBEC) ALI cultures. **(a)** Unique gene expression patterns of small airway epithelial markers SFTPB (n=6) and **(b)** large airway epithelial marker MUC5AC (n=6). SFTPB is significantly higher expressed in hSAEC compared to hBECs (p<0.0001). Additonally classical large airway marker is significantly higher expressed in hBEC compared to hSAEC, supporting the unique properties of the HTS adapted hSEAC (p<0.0022). **(c)** Comparison of CBF in hSAEC and hBEC (hSAEC n=36, hBEC n=12). In the here established system hSAEC show higher CBF values as to hBEC. In the boxplots, the horizontal line indicates the median, the box the interquartile range (25th to 75th percentiles) and the bars represent the range [min, max].

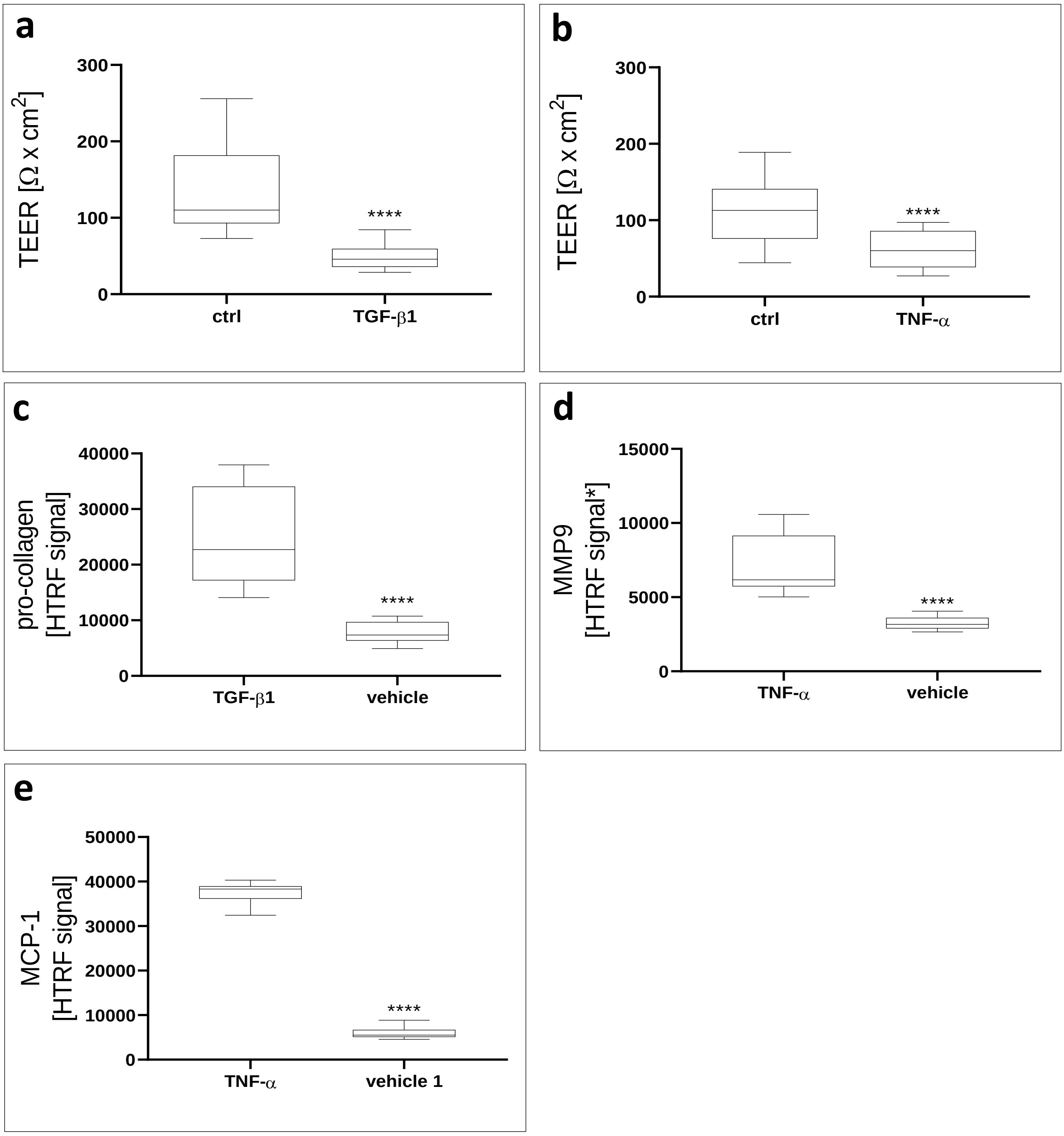

Figure S4. Changes in epithelial barrier function and pro-fibrotic marker expression of a different donor of hSEC cells (cat. CC-2547, Lot 548316) in the presence of different cytokines and respective inhibitors (n=16). (a,b) Breakdown of barrier function and subsequent loss in TEER, in the presence of 50 ng/mL TGF-β1 and 50 ng/mL TNF-α stimulation. **(c)** Pro-collagen I expression in the presence of TGF-β1 stimulation. **(d)** MMP9 expression in the presence of 50 ng/mL TNF-α stimulation. *Samples were pre-diluted 1:5 in DPBS. **(e)** MCP-1 expression in the presence of 50 ng/mL TNF-α stimulation.

Supplementary Tables

Table S 1 *Statistical details of one-sample t-test with a hypothetical value of 1, for qPCR expression levels.*

One-sample t-test	MUC5AC		MUC5B	
	24	96	24	96
Minimum	4,981	13,86	29,71	31,75
25% Percentile	6,619	15,26	31,55	41,41
Median	7,005	16,17	35,29	50,66
75% Percentile	7,512	16,52	37,55	53,53
Maximum	7,677	19,51	40,56	56,04
Range	2,696	5,646	10,85	24,3
Mean	6,856	16,2	34,96	47,7
Std. Deviation	0,8493	1,709	3,582	8,264
Std. Error of Mean	0,3003	0,6459	1,266	2,922
Theoretical mean	1	1	1	1
Actual mean	6,856	16,2	34,96	47,7
Number of values	8	7	8	8
One sample t test				
t, df	t=19,50, df=7	t=23,54, df=6	t=26,82, df=7	t=15,98, df=7
P value (two tailed)	<0,0001	<0,0001	<0,0001	<0,0001
P value summary	****	****	****	****
Significant (alpha=0.05)	Yes	Yes	Yes	Yes
Discrepancy	5,856	15,2	33,96	46,7
SD of discrepancy	0,8493	1,709	3,582	8,264
SEM of discrepancy	0,3003	0,6459	1,266	2,922
95% confidence interval	5,146 to 6,566	13,62 to 16,78	30,97 to 36,96	39,79 to 53,61
R squared (partial eta squared)	0,9819	0,9893	0,9904	0,9733

***Table S 2** Statistical details of one-sample t-test with a hypothetical value of 0, for qPCR expression levels and ciliary beating frequency.*

One-sample t-test	FOXJ1		CBF	
	24	96	24	96
Minimum	12,45	14,19	24	34
25% Percentile	13,1	14,88		
Median	13,47	16,76	5,255	5,295
75% Percentile	14,26	18,15	5,805	7,261
Maximum	15,84	18,46	6,209	8,648
Range	3,396	4,276	6,558	10,17
			7,036	11,49
Mean	13,7	16,5	1,781	6,2
Std. Deviation	1,038	1,571		
Std. Error of Mean	0,367	0,594	6,194	8,585
Theoretical mean	0	0	0	0
Actual mean	13,7	16,5	6,194	8,585
Number of values	8	7	24	34
One sample t test				
t, df	t=37,33, df=7	t=27,78, df=6	t=60,06, df=23	t=27,88, df=33
P value (two tailed)	<0,0001	<0,0001	<0,0001	<0,0001
P value summary	****	****	****	****
Significant (alpha=0.05)?	Yes	Yes	Yes	Yes
Discrepancy	13,7	16,5		
SD of discrepancy	1,038	1,571	6,194	8,585
SEM of discrepancy	0,367	0,594	0,5053	1,795
95% confidence interval	12,83 to 14,57	15,05 to 17,95	0,1031	0,3079
R squared (partial eta squared)	0,995	0,9923	5,981 to 6,408	7,959 to 9,212

Table S 3 *Statistical details of Mann-Whitney U-test for qPCR expression levels.*

Mann-Whitney U-test	KRT5		
	basal cells vs. 24	basal cells vs. 96	basal cells
Minimum	1,969	3,438	4,336
25% Percentile	2,17	4,054	4,336
Median	2,623	4,981	4,486
75% Percentile	2,89	6,023	5,658
Maximum	3,069	6,358	5,658
Range	1,1	2,92	1,322
Mean	2,561	4,973	4,827
Std. Deviation	0,3868	1,029	0,7239
Std. Error of Mean	0,1368	0,3639	0,4179
Mann Whitney test			
P value	0,0121	0,7758	
Exact or approximate P value?	Exact	Exact	
P value summary	*	ns	
Significantly different (P < 0.05)?	Yes	No	
One- or two-tailed P value?	Two-tailed	Two-tailed	
Sum of ranks in column A,C	36 , 30	50 , 16	
Mann-Whitney U	0	10	
Difference between medians			
Median of 24	2,623, n=8	4,981, n=8	
Median of basal cells	4,486, n=3	4,486, n=3	
Difference: Actual	1,863	-0,4945	
Difference: Hodges-Lehmann	2,156	-0,296	

Table S 4 *Statistical details of two-way ANOVA followed by an uncorrected Fisher's LSD test, for TEER data.*

Two-way ANOVA	TEER					
	24			96		
day	Mean	Upper Limit	Lower Limit	Mean	Upper Limit	Lower Limit
7	379,50	420,49	338,51	173,04	183,12	162,96
14	421,33	441,26	401,41	270,85	288,46	253,23
21	295,57	340,58	250,56	376,80	420,23	333,37
28	326,70	384,08	269,32	451,48	496,47	406,49
Two-way ANOVA	Ordinary					
Alpha	0,05					
Source of Variation	% of total variation	P value	P value summary	Significant?		
Interaction	6,879	<0,0001	****	Yes		
Row Factor	2,185	0,0406	*	Yes		
Column Factor	0,4794	0,175	ns	No		
ANOVA table	SS (Type III)	DF	MS	F (DFn, DFd)	P value	
Interaction	228387	3	76129	F (3, 169) = 8,876	P<0,0001	
Row Factor	72545	3	24182	F (3, 169) = 2,819	P=0,0406	
Column Factor	15914	1	15914	F (1, 169) = 1,855	P=0,1750	
Residual	1449491	169	8577			
Difference between column means						
Predicted (LS) mean of 24	355,8					
Predicted (LS) mean of 96	318					
Difference between predicted means	37,73					
SE of difference	27,7					

95% CI of difference	-16,95 to 92,42				
Number of rows (Row Factor)	4				
Number of values	177				
Number of families	1				
Number of comparisons per family	4				
Alpha	0,05				
Uncorrected Fisher's LSD	Predicted (LS) mean diff,	95,00% CI of diff,	Significant?	Summary	Individual P Value
24 - 96					
7	206,5	97,45 to 315,5	Yes	***	0,0003
14	150,5	41,62 to 259,4	Yes	**	0,007
21	-81,23	-191,1 to 28,63	No	ns	0,1463
28	-124,8	-234,5 to -15,03	Yes	*	0,0261

Table S 5 *Statistical details of two-way ANOVA followed by an uncorrected Fisher's LSD test, for Permeability data.*

Two-way ANOVA	Permeability								
	no cells			10 kDa, 24			10 kDa, 96		
minutes	Mean	Upper Limit	Lower Limit	Mean	Upper Limit	Lower Limit	Mean	Upper Limit	Lower Limit
0	59,13	60,57	57,68	59,13	60,01	58,24	46,75	47,46	46,04
15	10881,38	12174,44	9588,31	86,31	99,88	72,75	56,94	58,37	55,50
30	23015,00	26492,83	19537,17	117,88	139,46	96,29	76,69	79,84	73,53
45	33897,13	34706,41	33087,84	145,69	169,16	122,22	109,31	113,93	104,70
60	42741,63	44044,64	41438,61	204,44	245,49	163,38	159,88	168,89	150,86

Two-way ANOVA	Ordinary				
Alpha	0,05				
Source of Variation	% of total variation	P value	P value summary	Significant?	
Interaction	29,87	<0,0001	****	Yes	
Row Factor	18,99	<0,0001	****	Yes	
Column Factor	61,8	<0,0001	****	Yes	
ANOVA table	SS (Type III)	DF	MS	F (DFn, DFd)	P value
Interaction	7494448285	8	936806036	F (8, 185) = 1072	P<0,0001
Row Factor	4765098268	4	1191274567	F (4, 185) = 1364	P<0,0001
Column Factor	15505718408	2	7752859204	F (2, 185) = 8875	P<0,0001
Residual	161606384	185	873548		
Data summary					
Number of columns (Column Factor)	3				
Number of rows (Row Factor)	5				

Number of values	200
Number of families	5
Number of comparisons per family	2
Alpha	0,05

Uncorrected Fisher's LSD	Predicted (LS) mean diff,	95% CI of diff,	Significant?	Summary	Individual P Value
0					
no cells vs. 10 kDa, 24	-7,276E-12	-798,4 to 798,4	No	ns	>0,9999
no cells vs. 10 kDa, 96	12,37	-786,1 to 810,8	No	ns	0,9756
15					
no cells vs. 10 kDa, 24	10795	9997 to 11594	Yes	****	<0,0001
no cells vs. 10 kDa, 96	10824	10026 to 11623	Yes	****	<0,0001
30					
no cells vs. 10 kDa, 24	22897	22099 to 23696	Yes	****	<0,0001
no cells vs. 10 kDa, 96	22938	22140 to 23737	Yes	****	<0,0001
45					
no cells vs. 10 kDa, 24	33751	32953 to 34550	Yes	****	<0,0001
no cells vs. 10 kDa, 96	33788	32989 to 34586	Yes	****	<0,0001
60					
no cells vs. 10 kDa, 24	42537	41739 to 43336	Yes	****	<0,0001
no cells vs. 10 kDa, 96	42582	41783 to 43380	Yes	****	<0,0001

Table S 6 *Descriptive statistics for ELISA data.*

Descriptive Statistics	JAM-A	OCLN	CLDN1	TJP1
Number of values	8	8	8	7
Minimum	1,043	3,736	2,777	2,21
25% Percentile	1,521	4,097	3,516	2,806
Median	1,833	6,12	4,599	3,249
75% Percentile	2,089	7,086	5,18	4,257
Maximum	2,123	7,809	5,432	4,657
Range	1,081	4,072	2,655	2,447
Mean	1,771	5,732	4,336	3,399
Std. Deviation	0,368	1,556	0,9691	0,86
Std. Error of Mean	0,13	0,55	0,3426	0,325

Table S 7 *Statistical details of concentration-dependent stimulation experiments in hSAE cells.*

Nonlinear fit	Stimulation Experiments				
	TEER - TGF-β1	TEER - TNF-α	pro-collagen - TGF-β1	MMP9 - TNF-α	MCP-1- TNF-α
log(agonist) vs. response – Variable slope					
**Best-fit values**					
Bottom	11,52	-27,1	9721	4280	3989
Top	355,1	511,9	40013	27249	15731
LogEC50	-6,365	-4,77	-6,228	-4,842	-5,241
HillSlope	-1,67	-2,716	1,613	0,8032	0,9107
EC50	4,312E-07	0,00001699	5,911E-07	0,00001437	0,000005745
Span	343,6	539	30292	22968	11742
**95% CI (profile likelihood)**					
Bottom	-161,3 to 86,83	134,2	8219 to 11151	3962 to 4583	3763 to 4206
Top	316,1 to 396,4	467,1	36760 to 44569	23975 to 33874	14532 to 17622
LogEC50	-6,668 to -5,736	-5,025	-6,381 to -6,057	-5,028 to -4,511	-5,375 to -5,047
HillSlope	-0,5713	0,4904	0,9038 to 4,490	0,6481 to 0,9847	0,7348 to 1,118
EC50	2,146e-007 to 1,837e-006	9,448e-006	4,162e-007 to 8,760e-007	9,372e-006 to 3,082e-005	4,221e-006 to 8,979e-006
**Goodness of Fit**					
Degrees of Freedom	112	78	38	101	276
R squared	0,4472	0,4291	0,9202	0,9666	0,8704
Sum of Squares	2774144	2522782	481025866	105561116	449651460
Sy.x	157,4	179,8	3558	1022	1276
Number of points					
# of X values	144	96	78	288	288
# Y values analyzed	116	82	42	105	280

Table S 8 *Statistical details of concentration-dependent inhibition experiments in hSAE cells.*

Nonlinear fit	Inhibition Experiments		
	pro-collagen - TGF-β1 - SB431542	MMP9 - TNF-α - Ro 106-9920	MCP-1- TNF-α - Ro 106-9920
log(inhibitor) vs. response -- Variable slope			
**Best-fit values**			
Bottom	7906	5546	3035
Top	33665	43819	10362
LogIC50	-3,224	-3,127	-2,831
HillSlope	-1,402	-1,116	-1,292
IC50	0,0005968	0,0007468	0,001476
Span	25759	38274	7327
**95% CI (profile likelihood)**			
Bottom	5444 to 10053	179,4 to 9260	1880 to 3893
Top	32919 to 34429	42716 to 44953	10051 to 10687
LogIC50	-3,326 to -3,111	-3,259 to -2,937	-3,009 to -2,335
HillSlope	-1,887 to -1,038	-1,496 to -0,8270	-3,066 to -0,6488
IC50	0,0004721 to 0,0007752	0,0005506 to 0,001156	0,0009795 to 0,004626
**Goodness of Fit**			
Degrees of Freedom	128	260	260
R squared	0,8611	0,7356	0,5142
Sum of Squares	1449317539	12383677812	1138923180
Sy.x	3365	6901	2093
Number of points			
# of X values	132	264	264
# Y values analyzed	132	264	264

Table S 9 *Sampling approach and microscopic settings of the confocal z-stacks generated for the 24 and 96 Transwell ALI cultures.*

Scaling X	0,156 µm
Scaling Y	0,156 µm
Scaling Z	0,600 µm
Dimensions	x: 1024, y:1024, z: 60, channels: 4, 16-bit
Topography	High Qualitiy Mode not available
Image size	x: 160.04 µm, y: 160.04 µm, z: 35.40 µm
Scan Mode	stack
Slices	60 à 0.6 µm
Zoom	1.0
Objective	Plan-Apochromat 40x/1.4 Oil DIC M27
Position (x,y,z)	Position 1 x:11231.4 µm, y: -3578.2 µm, z: 150.4 µm
Pixel dwell	6.30 µs
Average	line 2
Master gain	Track 1 Ch1 : 514
	Track 2 Ch2 : 700
	Track 3 Ch3 : 500
	Track 4 Ch4 : 700
Digital gain	Track 1 Ch1 : 1.00
	Track 1 Ch2 : 1.00
	Track 1 Ch3 : 1.00
	Track 1 Ch4 : 1.00
Digital offset	Track 1 Ch1 : 0.00
	Track 1 Ch2 : 0.00
	Track 1 Ch3 : 0.00
	Track 1 Ch4 : 0.00
Pinhole	Track 1 Ch1 : 1.2 µm
	Track 1 Ch2 : 1.2 µm
	Track 1 Ch3 : 1.2 µm
	Track 1 Ch4 : 1.2 µm
Filters	Track 1 Ch1 : 0-1000
	Track 1 Ch2 : BP 505-600
	Track 1 Ch3 : 0-630
	Track 1 Ch4 : 0-514
Beam splitters	Track 1 MBS 405/488/555/639
	Track 1 1000 nm
	Track 2 MBS 405/488/555/639
	Track 2 559 nm
	Track 3 MBS 405/488/555/639
	Track 3 630 nm
	Track 4 MBS 405/488/555/639
	Track 4 468 nm
Lasers	Track 1 639 nm : 2.0%
	Track 2 555 nm : 5.0%
	Track 3 488 nm : 2.0%
	Track 4 405 nm : 2.0%

Supplementary Videos

Video S1. Z-stack of polarized and fully differentiated hSAE cells 4 weeks post-airlift cultured under HTS adapted conditions in 96-transwells.

Video S2. Synchronized ciliary beating of fully differentiated hSAE cells 4 weeks post-airlift cultured under HTS adapted conditions in 96-transwells.

Employed Software

GraphPad Prism version 8.0.0 for Windows, GraphPad Software, www.graphpad.com.

Microsoft PowerPoint 2016 (16.0.4266.1001) MSO (16.0.4993.1002) 32-Bit https://www.microsoft.com/de-de/microsoft-365/powerpoint.

ZEN 2.3 (blue edition), Carl Zeiss Microscopy GmbH, 2011 https://www.zeiss.de/mikroskopie/produkte/mikroskopsoftware/zen.html.

SOLIDWORKS 2017 https://www.3ds.com/de/produkte-und-services/solidworks/.

Supplementary References

1 Srinivasan, B. *et al.* TEER Measurement Techniques for In Vitro Barrier Model Systems. *Journal of Laboratory Automation* **20**, 107-126, doi:10.1177/2211068214561025 (2015).
